# A Novel Methicillin-Resistant* Staphylococcus aureus* t11469 and a Poultry Endemic Strain t002 (ST5) Are Present in Chicken in Ebonyi State, Nigeria

**DOI:** 10.1155/2017/2936461

**Published:** 2017-11-19

**Authors:** Amos Nworie, Azi S. Onyema, Simon I. Okekpa, Michael O. Elom, Nse O. Umoh, Victor U. Usanga, Gideon A. Ibiam, Boniface N. Ukwah, Lynda C. Nwadi, Chinwe Ezeruigbo, Busayo O. Olayinka, Joseph O. Ehinmidu, Josiah A. Onaolapo, Blake M. Hanson, Shylo E. Wardyn, Tara C. Smith

**Affiliations:** ^1^Department of Medical Laboratory Science, Faculty of Health Sciences, Ebonyi State University, Abakaliki, Nigeria; ^2^Department of Pharmaceutics and Pharmaceutical Microbiology, Faculty of Pharmaceutical Sciences, Ahmadu Bello University, Zaria, Nigeria; ^3^Department of Epidemiology, University of Iowa, Iowa City, IA, USA; ^4^Center for Emerging Infectious Diseases, University of Iowa, College of Public Health, Iowa City, IA, USA; ^5^Department of Nursing Science, Faculty of Health Science, Ebonyi State University, Abakaliki, Nigeria; ^6^Department of Biostatistics, Environmental Health Sciences, and Epidemiology, Kent State University, Kent, OH, USA

## Abstract

**Background:**

The changing epidemiology of methicillin-resistant* Staphylococcus aureus* (MRSA) from a hospital-associated pathogen to an organism commonly found in the community and in livestock reflects an organism well-equipped to survive in diverse environments and adjust to different environmental conditions including antimicrobial use.

**Methods:**

We investigated the molecular epidemiology of* S. aureus* and MRSA in poultry in Ebonyi State, Nigeria. Samples were collected from 1800 birds on 9 different farms within the state. Positive isolates were tested for antibiotic susceptibility and molecular typing.

**Results:**

Prevalence in birds was 13.7% (247/1800). MRSA prevalence in poultry was 0.8%. The prevalence of MRSA in broilers and layers was 1.2% and 0.4%, respectively. All tested isolates were susceptible to vancomycin. Molecular analysis of the isolates revealed 3* spa* types: t002, t084, and a novel* spa* type, t11469. The novel* spa* type t11469 belonged to sequence type ST5.

**Conclusion:**

The detection of t002 in chicken suggests the presence of livestock-associated MRSA in poultry in Ebonyi State. The detection of the new* spa* type t11469 in poultry that has not been characterised to ascertain its pathogenic potential remains a cause for concern, especially as some were found to carry PVL genes, a putative virulence factor in staphylococcal infection.

## 1. Introduction

*Staphylococcus aureus* is a known commensal of both man and animals and methicillin-resistant* Staphylococcus aureus* (MRSA) remains a major hospital and community pathogen [1–3]. Diseases associated with* S. aureus* include osteomyelitis, pneumonia, meningitis, arthritis, endocarditis, septicemia, deep tissue abscesses, skin, and soft-tissue infections (SSTIs), as well as toxic shock syndrome, among others [1, 4, 5].* S. aureus* is also a common cause of wound and urinary tract infections [6, 7].

*S. aureus* has been detected in several species of animals and products from animals [8–12], including poultry. The cloacae and nostrils of poultry remain important sites for the recovery of* S. aureus* and MRSA [13]. Intensity of contact with live animals is documented a risk factor for MRSA colonization [14]. While colonization alone does not harm the host, livestock-associated MRSA has also been reported to cause severe infections in humans, including endocarditis [15, 16]. However, while studies have been carried out investigating livestock-associated* S. aureus* in Europe, North America, and Asia, relatively few studies have been carried out in Africa. This study investigated the presence of* S. aureus*, including potential livestock-associated types, in poultry in Ebonyi State, Nigeria.

## 2. Materials and Methods

### 2.1. Sample Collection and Culture

Cloacae and nasal samples from 1800 birds (900 broilers and 900 layers) were collected using sterile swabs. Birds were selected at random from 9 poultry farms in the 3 senatorial districts in Ebonyi State, Nigeria (3 farms from each senatorial district). Swabs were processed within 24 hours by inoculation into 6.5% NaCl Staph enrichment broth. Isolation and identification of* S. aureus* were achieved through the use of mannitol salt plates, CNA plates with 5% sheep blood, and ChromAgar plates (CHROMagar, France). Isolates were confirmed as* S. aureus* using catalase and coagulase tests and further confirmed by Staph Latex Agglutination test (Pastorex Staph-plus, Bio Rad). Animal owners consented to testing of farm-owned poultry.

### 2.2. Molecular Testing

*S. aureus* isolates were tested for the Panton-Valentine leukocidin (PVL) genes, methicillin resistance gene (*mec*A), and staphylococcal protein A (*spa*) gene as previously described [17]. Multilocus sequence typing (MLST) was carried out as described by Enright et al. [18] and the detection of the tetracycline resistance gene tetM as described by Weigel et al. [19], and SCC*mec* typing was performed according to [20].

### 2.3. Antibiotic Susceptibility Testing

The resistance of isolates to a panel of 11 antibiotics was determined by the Kirby-Bauer-Clinical and Laboratory Standards Institute (CLSI) modified disc agar diffusion (DAD) method [21]. Isolates were tested for resistance to the following antibiotics: augmentin, cefoxitin, cefuroxime, chloramphenicol, doxycycline, erythromycin, gentamycin, levofloxacin, tetracycline, trimethoprim/sulfamethoxazole, and vancomycin.

### 2.4. Statistical Analysis

The data obtained were analyzed by ANOVA using the statistical package for social sciences (SPSS) version 20.2, Chicago, Illinois, USA. A *p* value of 0.05 or less was considered statistically significant.

## 3. Results

### 3.1. Prevalence of* S. aureus*

A total of 1800 birds consisting of 900 broilers and 900 layers from 9 farms in Ebonyi State were swabbed.* S. aureus* was recovered from the cloacae and nostrils of both broilers and layers. The number of broilers and layers positive for* S. aureus* was 122 (13.5%) and 125 (13.8%), respectively. The overall prevalence of* S. aureus* was 247/1800 (13.7%); see Table 1. Of the birds where* S. aureus* was recovered, 52.2% (129/247) of the colonized birds were positive in both the cloacae and nostrils, while 27.1% (67/247) were positive only in the cloacae, and 20.6% (51/247) of the colonized birds were positive only in the nostrils.

The percentage prevalence of broilers and layers varied between farms but were similar overall. Total prevalence ranged from 11.0% to 16.5%, in broilers, from 10.0% to 19.0%, and in layers, from 8.0% to 19.0% (Table 1).

Statistical analysis using ANOVA of birds with* S. aureus* in both the cloacae and nostrils, cloacae alone, and nostrils alone showed that there was a significant difference between the groups (*p* ≤ 0.05). Examining the prevalence of* S. aureus* recovered from broilers and layers showed a *p* value of 0.85, which was not significant.

### 3.2. Antibiotic Resistance

Antibiotic susceptibility testing (AST) of all the isolates (*n* = 247) was carried out against a panel of 11 antibiotics. Results showed that* S. aureus* exhibited varying degrees of susceptibility (Table 2). Over 40% of all isolates were resistant to tetracycline (45.7% of isolates tested, 113/247 isolates) and trimethoprim/sulfamethoxazole (40.9%; 101/247 isolates), almost 20% to erythromycin (19.4%, 48/247), and more than 10% to chloramphenicol (12.1%, 30/247). Fewer samples were resistant to doxycycline, cefoxitin, cefuroxime, gentamycin, and levofloxacin. No resistance was seen to vancomycin.

MRSA was recovered from multiple farms. The overall prevalence of MRSA was 15/1800 (0.8%) birds, or 6.1% of all positive isolates (15/247). MRSA was more common in broilers (11/900, 1.2%) compared to layers (4/900, 0.4%), a statistically significant difference. MRSA ranged from 0.0% (farms 4, 8, and 9) to 21.1% of positive broiler isolates (4/19) and 5/33 isolates (15.2%) on farm 1.

### 3.3. Molecular Analyses

*spa* typing results of 30 isolates selected equally from the 9 farms identified 3* spa* types: t002, t084, and t11469. There was an overlap of the* spa* types between MSSA and MRSA (Table 3). Of the isolates typed, 56.7% (17/30) were t002 and 30.0% (9/30) were t084. Novel* spa* type t11469 accounted for the remaining 13.3% (4/30) of isolates. All* spa* types included more MSSA than MRSA. In* spa* type t002, 70.6% of isolates of this* spa* type were MSSA (12/17), while 29.4% (5/17) were MRSA. In* spa* type t084, the prevalence of MSSA was 77.8% (7/9) and MRSA 22.2% (2/9). For* spa* type t11469, 75.0% (3/4) isolates were MSSA and 25.0% (1/4) were MRSA.

The 30* spa*-typed isolates were screened for PVL and* tetM*; 20.0% (6/30) were PVL-positive; 3.3% (1/30) were positive for* tetM* (*spa* type t084, MSSA). SCC*mec* analyses were carried out on 6 MRSA samples (3 from t002, 2 from t084, and one t11469). All the MRSA tested were* mecA* positive and were SCC*mec* type V. MLST analyses showed t084* spa* types belonged to ST15 and t11469 to ST5, and t002* spa* types were a mix of ST5, ST15, and ST121.

## 4. Discussion

*S. aureus* and methicillin-resistant* S. aureus* were recovered from both broilers and layers in this study. The percentage of animals with* S. aureus* recovered from both cloacae and nostrils were higher than from cloacae and nostrils alone. This suggests that cloacae and nostrils are important sites for the recovery of* S. aureus*, a combination which gives better yield. This was consistent with earlier work in Belgium [13], which reported higher recovery of* S. aureus* from multiple sites than the sites screened singly.

All the isolates exhibited varying degrees of resistance to the panel of 11 antibiotics tested. Almost half (45.7%) of tested samples were resistant to tetracycline. Tetracycline is the most commonly used food supplement and growth promotion factor in poultry farms in this area. Others include trimethoprim/sulfamethoxazole, chloramphenicol, erythromycin, and quinolones. These drugs are also used for disease treatment in these farms. Regulation of antibiotic use in poultry in Ebonyi state and Nigeria more broadly is lacking, which could contribute to the high levels of resistance observed in this environment.

Of 30 isolates examined, only 1 (3.3%) was positive for the* tetM* gene, one of the genes encoding for tetracycline resistance. The low percentage of* tetM* recorded might suggest the involvement of other tetracycline resistance genes in tetracycline resistance observed in this study. Fluit et al. [22] have reported that tetracycline resistance is determined by several tetracycline (*tet*) genes, and the involvement of other tetracycline resistance genes in poultry has been reported elsewhere [23].

The percentage recovery of MRSA from broilers was 1.2% and  .4% in layers. Reasons for the differences in the recovery of MRSA from broilers and layers were not clear, as their rearing conditions were similar. Though there was no significant difference between number of* S. aureus* recovered from broilers and layers, there was a significant difference in MRSA recovered from both, though this was a comparison of small numbers and caution should be taken not to overinterpret results.

*spa* typing showed that 3* spa* types of* S. aureus*, including a livestock-indicator* spa* type t002, were circulating among chicken in Ebonyi State. Lowder et al. [12] and Köck et al. [16] previously reported this* spa* type in poultry. A novel* spa* type t11469 was also detected, which to the best of our knowledge has never been characterised in Ebonyi or elsewhere. Our data showed that 6 isolates were positive for PVL gene, out of which 5 were MSSA while one was MRSA. The finding of PVL-carrying isolates in chicken may portend a serious health hazard to the poultry farm workers and their families due to possibility of farm-to-family transmission and the association of PVL with heightened virulence, though this remains controversial [24]. Furthermore, the isolation of* lukS-lukF* carrying isolates of* S. aureus* in this study might constitute a health risk to the general public as chicken are reared in homes and live chicken are sold on the streets and in rural markets where little attention is paid to hygiene. Prior reports have shown that PVL-positive MSSA is common in West Africa [25].

Multilocus sequence analysis showed that* S. aureus* in chicken in Ebonyi State displayed three sequence types: ST5, ST15, and ST121. ST5 had 2* spa* types: t002 and t11469. Surprisingly, t002 was found in all three identified sequence types. The* spa* type t002 of ST5 is a livestock endemic strain thought to have left its niche as a nosocomial strain to livestock from where it is involved in human diseases [12], similar to that of another livestock-associated strain, ST398 [26]. The documentation of the novel strain, t11469, in ST5 could suggest that it could be related to t002; however, their* spa* repeats are quite different (t002, 26-23-17-34-17-20-17-12-17-16; t11469, 04-44-24-33-31-12-16-34-12-25-22-34), suggesting divergent* spa* genes.

The discovery of* spa* type t002 in ST15 and ST121 was intriguing, as available reports have not demonstrated t002 in these sequence types. Its presence in these sequence types could either suggest adaptation to different ecological condition, as previously reported [27], or local dispersion of* spa* types in different regions and environment [28], for example, by horizontal transmission of the* spa* gene into genetic backgrounds of ST15 and ST121 strains. Furthermore, the recovery t084 in poultry suggests an organism highly equipped to cross interspecies borders. It could also be suggestive of the movement of MRSA from its niche in human clinics to livestock as has been variously reported [29]. All MRSA isolates tested carried SCC*mec* type V, which has been found both in the community [30] and in livestock, including ST398 in pigs [8, 15].

There were important limitations to this study. Though birds within the farms were randomly selected, a convenience sample of farms participated, potentially biasing the study. However, it is unlikely that farmers had any knowledge of the* S. aureus* status of their farms prior to enrolment. Samples from individuals or the environment were not collected, and as budgetary issues allowed only a subset of positive samples to be molecularly typed (30 of 247 positive isolates), we may have missed some of the diversity in our broader sample. Further research studies should examine birds within live markets as well as on farms in order to assess risk to consumers in addition to farm workers.

## 5. Conclusion

The detection of a new* spa* type t11469 carrying the PVL gene could portend a health hazard if it is commonly transmitted to humans. The isolation of this new* spa* type in poultry especially as it has not been characterized to establish its pathogenic potential or its inherent virulence factors remains a public health threat. Additional sequencing and characterization of t11469 are necessary to establish the pathogenic potential of this novel* spa* type. Poultry farm workers in this environment should ensure the highest level of hygiene, including constant hand washing to prevent interspecies transfer of this new strain, or other strains of* S. aureus* that may be present on Nigerian poultry farms.

## Figures and Tables

**Table 1:** Percentage prevalence of *S. aureus* in broilers and layers per farm.

Farm #	Layers, # (%) positive	Broilers, # (%) positive	Overall, # (%) positive
Farm 1	19 (19.0%)	14 (14.0%)	33 (16.5%)
Farm 2	13 (13.0%)	18 (18.0%)	31 (15.5%)
Farm 3	11 (11.0%)	19 (19.0%)	30 (15.0%)
Farm 4	11 (11.0%)	12 (12.0%)	23 (11.5%)
Farm 5	17 (17.0%)	8 (8.0%)	25 (12.5%)
Farm 6	10 (10.0%)	13 (13.0%)	23 (11.5%)
Farm 7	19 (19.0%)	16 (16.0%)	35 (17.5%)
Farm 8	12 (12.0%)	10 (10.0%)	22 (11.0%)
Farm 9	10 (10.0%)	15 (15.0%)	25 (12.5%)

Total	122 (12.2%)	125 (12.5%)	247 (13.7%)

100 broilers and 100 layers were sampled on each farm.

**Table 2:** Antibiotic resistance of poultry isolates.

Antibiotic	*#* (%) resistant
Augmentin	2 (0.8%)
Cefoxitin	15 (6.1%)
Cefuroxime	13 (5.3%)
Chloramphenicol	30 (12.1%)
Doxycycline	19 (7.7%)
Erythromycin	48 (19.4%)
Gentamycin	13 (5.3%)
Levofloxacin	2 (0.8%)
Tetracycline	113 (45.7%)
Trim/sulf	101 (40.9%)
Vancomycin	0 (0%)

**Table 3:** Molecular analyses of selected isolates.

*spa* type	MSSA # (%)	MRSA # (%)	PVL	*tetM*	Sequence type (ST)
t002	12 (70.6%)	5 (29.4%)	4 (MSSA)		ST 5, ST 15, ST 121
t084	7 (77.8%)	2 (22.2%)	1 (MSSA)	1 (MRSA)	ST 15
t11469	3 (75.0%)	1 (25.0%)	1 (MSSA)		ST5
